# Differing Time Courses of Reward-Related Attentional Processing: An EEG Source-Space Analysis

**DOI:** 10.1007/s10548-021-00827-3

**Published:** 2021-03-18

**Authors:** Denise E. L. Lockhofen, Nils Hübner, Fatma Hemdan, Gebhard Sammer, Dion Henare, Anna Schubö, Christoph Mulert

**Affiliations:** 1 Centre for Psychiatry and Psychotherapy, Justus-Liebig-University Giessen, Klinikstraße 36, 35385 Giessen, Hessen Germany; 2 Cognitive Neuroscience of Perception and Action, Faculty of Psychology, Philipps-University Marburg, Gutenbergstr. 18, 35032 Marburg, Germany

**Keywords:** Attentional selection, Top-down attention, Bottom-up attention, Reward, EEG, Source space analysis

## Abstract

Since our environment typically contains more information than can be processed at any one time due to the limited capacity of our visual system, we are bound to differentiate between relevant and irrelevant information. This process, termed attentional selection, is usually categorized into bottom-up and top-down processes. However, recent research suggests reward might also be an important factor in guiding attention. Monetary reward can bias attentional selection in favor of task-relevant targets and reduce the efficiency of visual search when a reward-associated, but task-irrelevant distractor is present. This study is the first to investigate reward-related target and distractor processing in an additional singleton task using neurophysiological measures and source space analysis. Based on previous studies, we hypothesized that source space analysis would find enhanced neural activity in regions of the value-based attention network, such as the visual cortex and the anterior cingulate. Additionally, we went further and explored the time courses of the underlying attentional mechanisms. Our neurophysiological results showed that rewarding distractors led to a stronger attentional capture. In line with this, we found that reward-associated distractors (compared with reward-associated targets) enhanced activation in frontal regions, indicating the involvement of top-down control processes. As hypothesized, source space analysis demonstrated that reward-related targets and reward-related distractors elicited activation in regions of the value-based attention network. However, these activations showed time-dependent differences, indicating that the neural mechanisms underlying reward biasing might be different for task-relevant and task-irrelevant stimuli.

**Supplementary Information:**

The online version contains supplementary material available at 10.1007/s10548-021-00827-3.

## Theory

Visual attention controls the way we search for information from the environment. Attentional selection is usually described in terms of bottom-up and top-down processes, whereby bottom-up processes are assumed to be controlled by the physical salience of events in the environment and top-down processes are thought to be under volitional control of the observer and thus dependent on internal goals, intentions and beliefs (Corbetta and Shulman 2002).

This dichotomy of bottom-up and top-down attention remained unchallenged for many years, but recent research suggests that there are additional factors influencing attentional control. One of these factors seems to be reward (Awh et al. 2012). Monetary reward can bias attentional selection in favor of task-relevant stimuli (Della Libera and Chelazzi 2006; Engelmann et al. 2009), presumably through enhanced preparation of strategic and task-related processes (Kiss et al. 2009). It can also reduce the efficiency of visual search when a task-irrelevant, reward-associated distractor is present (Anderson et al. 2013; Bourgeois et al. 2017; Chelazzi et al. 2013; Hickey et al. 2010; Le Pelley et al. 2015; Watson et al. 2019). In spite of these findings, there are still many open questions about the way reward affects the interplay of visual salience and voluntary attentional control, as well as concerning the underlying neurophysiological and neurobiological mechanisms.

A paradigm well suited to investigate the contributions of different attentional processes on attentional selection is the Additional Singleton Task (AST; Theeuwes 1991). It offers the advantage that rewards can be associated with stimuli that have not been previously related to the task, ruling out potential motivational effects (Le Pelley et al. 2015). The AST features different kinds of singletons, stimuli that stand out, because they differ from the other stimuli in the search display in a basic visual dimension (such as color or orientation). In the original AST by Theeuwes (1991), observers had to search for a salient singleton (e.g. green diamond shape) while ignoring a simultaneously presented irrelevant singleton (e.g. red circle). The author found that reaction times were slower when the colored distractor was present than when it was not.

Feldmann-Wüstefeld et al. (2016) used a variant of the AST to investigate an EEG-component, termed N2-posterior-contralateral (N2pc). The N2pc is an attention-sensitive event-related potential (ERP), elicited at post-stimulus latencies of 200 to 350 ms and typically associated with the distribution of spatial attention to a task-related stimulus (Luck and Hillyard 1994; Li et al. 2017). It can only be seen for laterally presented stimuli and refers to a more negative amplitude in posterior electrodes contralateral to the target compared with posterior electrodes ipsilateral to the target. In their study, Feldmann-Wüstefeld et al. (2016) found that high-reward distractors were more likely to capture attention (reflected by an increased N_D_ subcomponent of the N2pc) and harder to suppress (indicated by a higher P_D_ subcomponent of the N2pc) than low-reward distractors. These results suggest that reward might be particularly involved in increasing stimulus salience. Thus, neurophysiological measures underscore the role of reward as an important factor in determining attentional selection (Feldmann-Wüstefeld et al. 2016).

Another important step towards understanding the mechanisms underlying the impact of reward on attentional selection is the attempt to relate attentional processes to structures in the brain. Generally, it is assumed that bottom-up attention is associated with activation of temporoparietal and ventral frontal areas, whereas top-down attention is centered on the dorsal posterior parietal and frontal cortex (Corbetta and Shulman 2002). Concerning attentional capture, functional magnetic resonance imaging (fMRI) showed that interference from salient, but task-irrelevant distractors increased activity in superior parietal and frontal regions (de Fockert et al. 2004; Lavie et al. 2006). When task-irrelevant, but previously reward-associated stimuli were presented, extrastriate visual areas, the intraparietal sulcus and parts of the basal ganglia were involved, forming a network responsible for value-based attentional selection (Anderson et al. 2014, 2017). In addition, reward was also found to modulate stimulus salience via the anterior cingulate (Hickey et al. 2010). However, there is yet no study integrating neurophysiological findings and brain localization methods in an Additional Singleton Task with reward-related targets and distractors. This integration would be especially beneficial, since it makes use of the high temporal resolution of the EEG, allowing for a better temporal delineation of neural events that cannot be achieved by other localization techniques (e.g. fMRI) alone.

Therefore, to the best of our knowledge, the current study was the first to combine ERP analysis and source localization during reward-related target and distractor processing. In contrast to Feldmann-Wüstefeld et al. (2016) we not only investigated the processing of reward-related distractors, but employed two participant groups: one in which the distractor was rewarded and one in which the target was rewarded. By introducing a target-reward group, we were able to examine how reward magnitude modulated attentional selection when task-relevant targets were associated with reward compared to when task-irrelevant distractors were associated with reward. Associating targets and distractors with reward was achieved during the experiment to make sure distractors did not have any previous relevance to the task.

To disentangle target and distractor processing in the EEG, we used a systematic lateralization technique (Hickey et al. 2009; Woodman and Luck 2003). This technique takes advantage of the contralateral organization of the visual system in order to isolate the processing of a certain stimulus in the event-related potential. Stimuli presented in the right visual field are usually processed in the contralateral left hemisphere of the cortex, and vice versa. By presenting a stimulus of interest sometimes in the left visual field and sometimes in the right visual field, it is possible to take the contralateral neural response on each trial and subtract its equivalent ipsilateral response. Therefore, in the averaged lateralized ERP we have isolated activity that is systematically related to the stimulus of interest. Crucially for our paradigm, any stimulus that is presented on the midline of the visual display will be processed equally in the left and right hemisphere and will, therefore, be removed from the lateralized ERP during subtraction. In this way, we can isolate distractor processing with trials where the target is placed on the midline while the distractor is lateralized, and isolate target processing by including trials with the reverse arrangement. Previous work (Feldmann-Wüstefeld et al. 2016) has shown that targets elicit a lateralized negativity (N_T_) that is associated with target prioritization. Distractors, however, elicit both a negativity (N_D_), reflecting attentional capture by the distractor, and a positivity (P_D_) that is believed to reflect active suppression of the distractor. We assumed that high rewards would lead to an enhanced capture of attention in the distractor reward group (reflected by increased N_D_/P_D_ amplitudes) and an enhanced target prioritization in the target reward group (reflected by increased N_T_ amplitudes).

In addition to ERP analysis, we employed source localization. We hypothesized that reward-related targets and distractors would enhance brain activity in parietal and frontal regions associated with attention (de Fockert et al. 2004; Lavie et al. 2006) and in areas previously found to be involved in reward processing, such as the extrastriate visual cortex and the anterior cingulate (Anderson et al. 2014, 2016, 2017; Hickey et al. 2010). Going further, we took advantage of the high temporal resolution of the EEG and examined the timeline of neural activation associated with reward-related attentional processing. Since this was the first time source analysis has been used to investigate the time course of reward effects on targets and task-irrelevant distractors, we pursued a more exploratory approach.

## Method

### Ethics Statement

The study was approved by the Institutional Review Board of the University of Giessen and carried out in accordance with the latest version of the Declaration of Helsinki. Written informed consent was obtained from all participants.

### Participants

Forty volunteers (20 male, mean age = 25 years, SD = 4.5, range 18–35; 21 female, mean age = 25 years, SD = 4.9, range 21–39) participated in the experiment. All were right-handed and had normal or corrected to-normal vision and no color-blindness (assessed with the Snellen Vision Test and the Ishihara Test for Color Deficiency). Individuals with a history of neurological or psychiatric disease were excluded. Participants were told that correct responses would yield them points and that an equivalent amount of these points would be paid after the experiment in Euros. 1000 points corresponded to a reward of 4.19 EUR and 100% correct responses would yield the participant a total of 5808 points (24.39 EUR). Since participants performed well, none of them earned less than 20 EUR. All participants gave their informed written consent to participate in the study. One participant had to be excluded because of technical difficulties.

### Stimuli and Apparatus

The experiment was performed in a dimly lit, electrically shielded room. Participants were asked to sit in a comfortable chair and to respond to the stimuli orientation by pressing one of two response-buttons on a three-button device with the index finger of their dominant hand (one button for leftward-tilted targets and one button for rightward-tilted targets) while holding a hold-button in between responses. Stimuli were presented using Presentation (Version 20.1, Neurobehavioral Systems Inc.) on an LCD screen (Asus VZ249HE-W; 23.8″ screen diagonal), located 100 cm from the participant. Based on the experiment from Feldmann-Wüstefeld et al. (2016), the search display was arranged as a 27 × 17 matrix (20° × 13° of visual angle) of 458 light-grey (RGB: 134, 134, 134) line elements, presented on a dark grey (RGB: 60, 60, 60) background with a fixation dot in the center. Individual line-elements had a length of 0.7° of visual angle and were either horizontal (50%) or vertical (50%). While the target was also a light grey line-element, tilted 45° to the right or to the left, the distractor was a blue (RGB: 82, 124, 255) or red (RGB: 232, 34, 34) line-element, randomly chosen to be horizontal or vertical. Within the matrix, target and distractor were always presented at two out of six fixed positions. When the target was presented laterally (in half of the trials), the distractor was presented on a vertical midline position, and when the distractor was presented laterally (in the other half of the trials), the target was presented on a vertical midline position. These vertical midline positions were 4.6° above and below the fixation dot. The lateral positions were 3.8° left and right of the vertical midline and 2.3° above and below the horizontal midline. The other matrix positions were filled randomly with horizontal and vertical line-elements.

**Fig. 1:**
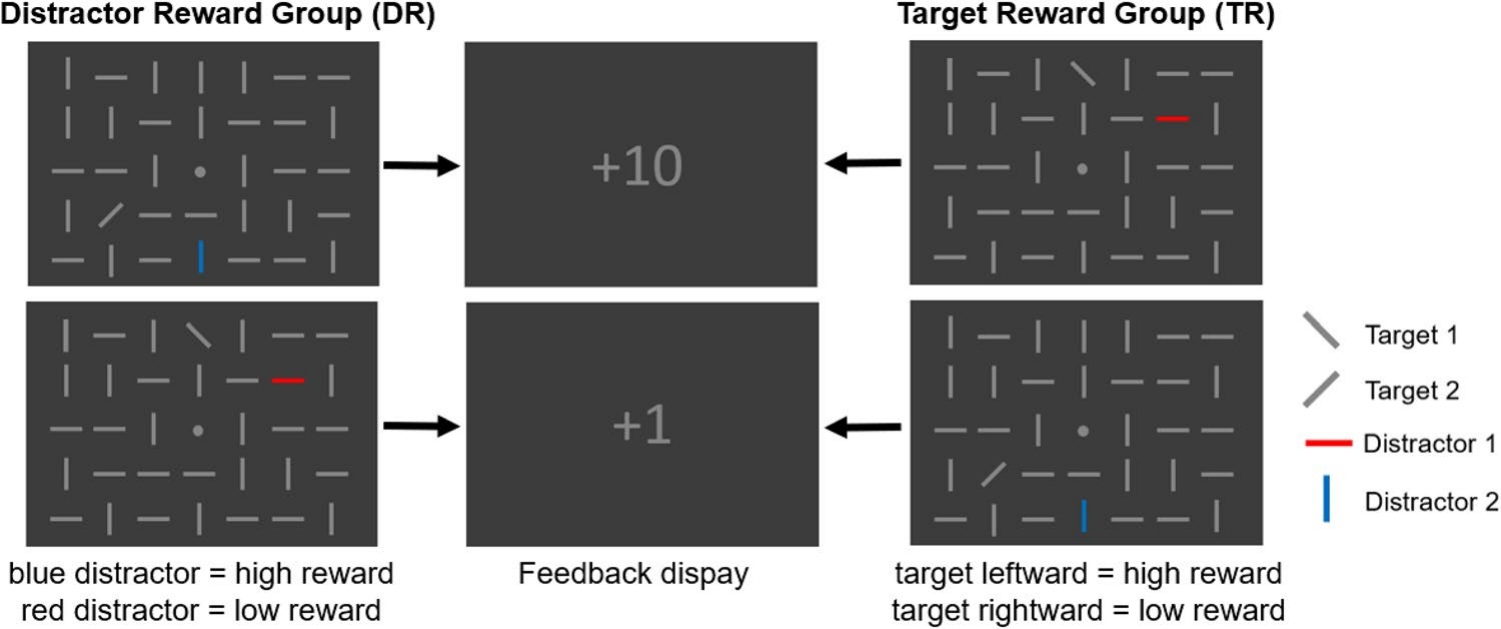
An illustration of the search display used in the experiment (with a reduced number of line-elements). The task of the participant was to respond to the target orientation by button press. The assignment of button position (left or right) to target orientation (leftward-tilted or rightward-tilted) was counterbalanced across participants. In the DR condition (left), high and low rewards were tied to distractor color, and in the TR condition (right), high and low rewards were tied to the target orientation. The feedback display showed + 10 for correct responses in high reward trials, + 1 for correct responses in low reward trials and + 0 for incorrect responses

There were two participant groups (see Fig. 1). For one half of the participants (n = 20) reward was tied to the color of the distractor (red or blue, distractor reward group, DR) and for the other half (n = 20) reward was associated to the direction of the target (left or right, target reward group, TR). Thus, participants were rewarded depending on the accuracy of their response, but the magnitude of their reward (high or low) was dependent on distractor color (DR) or target orientation (TR). Within the two groups, trials with red and blue distractors (DR) or leftward-tilted and rightward-tilted targets (TR) were presented equally often. In the DR group, the color blue was linked to high rewards for one half of the subjects (n = 10) and with low rewards for the other half (n = 10). Likewise, in the TR group, leftward-tilted targets were tied to high rewards for one half of the subjects (n = 10) and with low rewards for the other half (n = 10). About 27% of trials in each group were target-only trials, in which no distractor was presented. For these trials, the reward condition (high or low) was chosen randomly (in the DR group).

### Procedure

Prior to the experiment, participants were given the opportunity to train the experiment. Therefore, one block of the task was presented as training block. The training was repeated twice, first with a slower version of the task, so that participants could get accustomed to the display and the handling of the button-device, and then with the task at original speed. Participants were informed that rewards obtained during the training would not be transferred to the experiment. Thus, each experiment started with a bank account of zero.

At the beginning of each trial, a central fixation dot was presented for 500 ms, followed by the search display for 200 ms, which was subsequently replaced by a central fixation dot for 1400 ms or until the response of the participant. Responses slower than 1400 ms were automatically counted as incorrect. Afterwards, a blank screen was presented for 100 ms, followed by the feedback display for 800 ms and another blank screen for 800 ms. The feedback display showed + 10 for correct responses in high reward trials and + 1 for correct responses in low reward trial and + 0 for incorrect responses.

The main factors of the experiment were the between-subjects-factor reward group (distractor reward vs. target reward) and the within-subjects-factors target laterality (lateral position vs. central position) and distractor color (red vs. blue, DR) or target orientation (tilted to the left vs. tilted to the right, TR). These factors, together with actual target/distractor location (position in the matrix) were controlled for in the experiment. While doing so, target and distractor position were chosen to always be on the same side of the horizontal midline. Thus, there were 32 possible factor combinations in total and four combinations of the main factors (laterality and distractor color/target identity). One of these combinations was randomly chosen in each trial and each combination of the main factors was used 192 times per subject. Together with the target-only trials (288 per subject), this resulted in a total number of 1056 trials, divided into 24 blocks of 44 trials (32 normal trials + 12 target-only trials).

After each block, participants received feedback regarding their performance. They were shown their averaged response times and accuracy, as well as an account balance, listing their total amount of points and money (in Euros). Since participants were not informed about the reward scheme, they were presented with a post-experiment questionnaire asking them if they saw the distractor (yes/no) and realized that the reward was associated to the color of the distractor/orientation of the target (yes/no). Additionally, participants were asked to complete several questionnaires (Schizotypal personality questionnaire, SPQ, Raine 1991; Cardiff Anomalous Perception Scale, CAPS, Bell 2006).

### EEG Recording

Electroencephalographic activity was recorded at a sampling rate of 1000 Hz with 64 Ag/AgCl electrodes mounted on an elastic cap (ActiCaps, Brain Products, Munich, Germany), using the Brain Vision Recorder software version 1.21.0303 (Brain Products, Munich, Germany). Electrodes were arranged according to a modified 10/20 system without electrodes at positions FPz, F9, F10, CP3, CP4, P9, P10, PO7, PO8, and with two additional electrodes at positions PO9 and PO10. Eye movements were recorded with four EOG channels (positioned at the outer canthi bilaterally and infra- and supraorbitally on the right). An electrode at the FCz position was used as reference, while the electrode at position AFz served as ground. Electrode impedances were always kept below 5 kΩ.

### Data Analysis

#### Behavioral Data

Median response times were calculated for each participant, separately for each reward condition (high or low reward). Median was used because it is more robust regarding potential outliers. Trials with incorrect responses were removed. The remaining data was submitted to a 2 × 2 analysis of variance (ANOVA) with the between-subjects factor group (DR or TR) and the within-factor reward condition (high or low reward). Additionally, paired t-tests were calculated to test for differences between normal and target-only trials.

#### EEG Data Preprocessing

Data analysis was done using the Brain Vision Analyzer software version 2.2 (Brain Products, Munich, Germany). The data was re-referenced to common average without EOGs. After band-pass filtering the EOG channels (High cut off 15 Hz), all data sets were corrected for eye movement and blink artifacts by applying independent component analysis. Subsequently, the continuous EEG was filtered (Low cut off 0.5 Hz, High cut off 20 Hz, Notch Filter 50 Hz) and then segmented into 700-ms epoch including a 200-ms prestimulus baseline. Trials with incorrect responses were excluded from further analysis. During artifact rejection, amplitudes exceeding ± 50 μV or activity lower than 0.1 μV were automatically rejected. Afterwards, a baseline correction (using an interval of 200 ms prior to the stimulus) was applied.

#### Calculation of Lateralized ERPs

In order to disentangle target and distractor related processing and to calculate subcomponents of the N2pc, we used a systematic lateralization technique (Hickey et al. 2009; Woodman and Luck 2003). We first calculated the mean lateralized ERPs by subtracting the activity ipsilateral to the target/distractor from the activity contralateral to the target/distractor). Target-only trials were not included in the analysis. For all remaining trials, we collapsed the mean lateralized ERPs of the electrode pairs PO3/P7 and PO4/P8 across reward conditions and determined epochs corresponding to the N2pc subcomponents. The analysis epoch for N_T_ was determined as ± 50 ms around to the first negative peak in the grand average for trials in which the targets were presented laterally and the distractors at the central midline position. Likewise, the analysis epoch for N_D_ was determined as ± 50 ms around the first negative peak in the grand average for trials in which the distractors were presented laterally and the targets at the central midline position. Finally, the analysis epoch for P_D_ was determined as ± 50 ms relative to the first positive peak after the N_D_. The N_T_ epochs were 175–275 ms for both DR and TR. The N_D_ epochs were 162–262 ms (DR) and 163–263 ms (TR). The P_D_ epochs were 238–338 ms (DR) and 228–328 ms (TR). Subsequently, mean lateralized ERPs were calculated for these epochs, separately for each group and reward condition. The resulting data was submitted to three 2 × 2 ANOVAs, one for each EEG component, with group (DR or TR) as between-subjects factor and reward condition (high or low reward) as within-subjects factor.

#### Source-Space Analysis

Source-space localization analyses were performed with low-resolution brain electromagnetic tomography (LORETA) KEY software package (http://www.uzh.ch/keyinst/loreta; Version 20200414; Nichols and Holmes 2001; Pascual-Marqui et al. 1994, 1999). Using LORETA it is possible to compute distributed activity throughout the brain based on a three-shell spherical head model registered to standard space (MNI brain) and restricted to cortical gray matter. LORETA operates under the smoothness-assumption (Pascual-Marqui et al. 1999), stating that neighboring neurons are simultaneously and synchronously active, and has been shown to provide source localization results in line with fMRI findings at relatively low spatial resolution (Mulert et al. 2004). In this study, we used the sLORETA (standardized low resolution brain electromagnetic tomography) method, which was shown to be robust against measurement and biological noise (Pascual-Marqui et al. 2002). Source localization was executed for the averaged, non-lateralized signal in DR and TR groups (averaged across participants and reward conditions). For the transformation matrix, we used automatic regularization and a spatial over-smoothing (signal-to-noise ratio of 10).

First, to investigate the time course of neural activity, a time window from 50 to 400 ms after stimulus onset was constructed and divided into epochs of 50 ms duration. Current source density values were obtained from the averaged signal during these epochs, separately for the DR and the TR group. Afterwards, DR and TR groups were directly compared within each 50-ms-epoch. Additionally, we compared high and low reward trials within the EEG epochs determined above (N_T_: 175–275 ms, N_D_: 162–262 ms (DR) and 163–263 ms (TR), P_D_: 238–338 ms (DR) and 228–328 ms (TR)). The voxel-wise comparison of cortical activities was done using two-tailed t-tests (statistical significance threshold *p* < 0.05) for independent (DR > TR) or paired (high reward > low reward) groups implemented in the LORETA software. A statistical nonparametric mapping randomization method was used to adjust for multiple comparisons with a Fisher's random permutation test with 5000 randomizations. This method corrects for multiple testing by estimating via randomization the empirical probability distribution for the max-statistic under the null hypothesis (Nichols and Holmes 2001).

## Results

### Behavioral Data

**Fig. 2:**
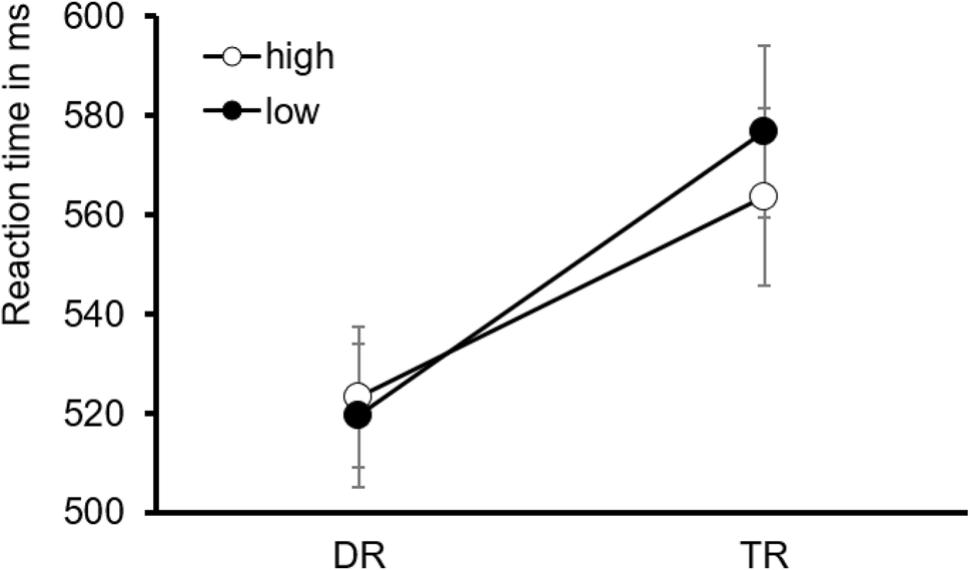
Interaction between group (DR, TR) and reward condition (high, low reward). Error bars denote the standard error of the mean

Analysis of response accuracy revealed that participants made less than 5% errors. Concerning the response times, the 2 × 2 ANOVA revealed a significant interaction between group and reward condition, *F*(1,38) = 5.874, *p* = 0.020, $\eta_{{\text{p}}}^{2}$ = 0.134 (Fig. 2). Thus, the effect of reward condition was different for DR and TR groups. Additionally, there was a significant between-subjects effect for group, *F*(1,38) = 4.721, *p* = 0.036, $\eta_{{\text{p}}}^{2}$ = 0.111. Contrasts revealed significantly faster reaction times for high-reward targets than for low-reward targets, *t*(38) = − 2.669, p = 0.011 in the TR group. Furthermore, in the DR group, paired t-tests showed a significant difference between target-only trials and high-reward trials, *t*(19) = 6.528, *p* < 0.025, and between target-only trials and low-reward trials, *t*(19) = 5.251, *p* < 0.025 (Bonferroni-corrected for two comparisons). In the TR group, paired t-tests showed a significant difference between high-reward target-only trials and high-reward trials, *t*(19) = 6.106, *p* < 0.025, and between low-reward target-only trials and low-reward trials, *t*(19) = 4.737, *p* < 0.025 (Bonferroni-corrected for two comparisons). Therefore, participants were significantly faster in trials without a distractor (Table 1).

**Table 1:** Descriptive statistics for DR and TR groups (response times in ms)

Reward	DR			TR			
	HR	LR	TO	HR	LR	TO-HR	TO-LR
N	20	20	20	20	20	20	20
Mean	524.2	520.0	511.5	563.6	576.6	549.2	564.5
Std. Dev	64.9	66.5	63.5	80.0	77.4	81.1	74.0
Max	658.0	650.1	413.1	746.6	751.3	452.8	465.7
Min	414.9	416.2	647.4	459.4	476.7	737.6	727.7

*HR* high reward, *LR* low reward, *TO* target-only, *DR* distractor reward, *TR* target reward, *Std.Dev*. standard deviation

### ERP Results

**Fig. 3:**
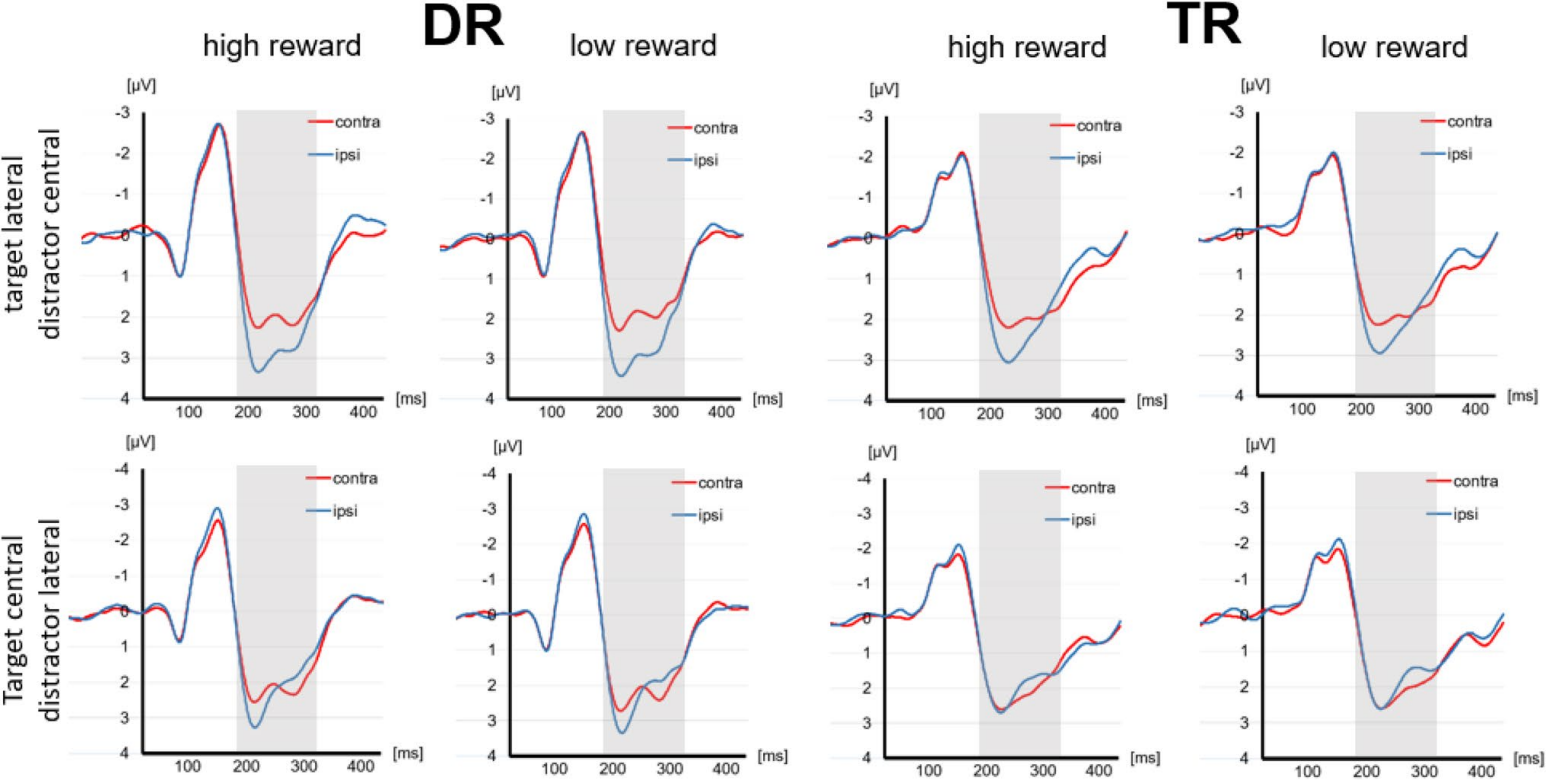
Grand average basic ERPs recorded in the distractor reward group (left) and in the target reward group (right) for high and low reward trials. ERPs were pooled over the electrode pairs PO3/P7 and PO4/P8. The upper row shows the contralateral (red) and ipsilateral (blue) ERPs evoked by the search displays, when targets were presented at a lateral position and distractors were presented at a central midline position. The lower row shows the contralateral (red) and ipsilateral (blue) ERPs evoked by the search displays, when targets were presented at a central midline position and distractors were presented at a lateral position. The areas representing the N2pc are marked (Color figure online)

**Fig. 4:**
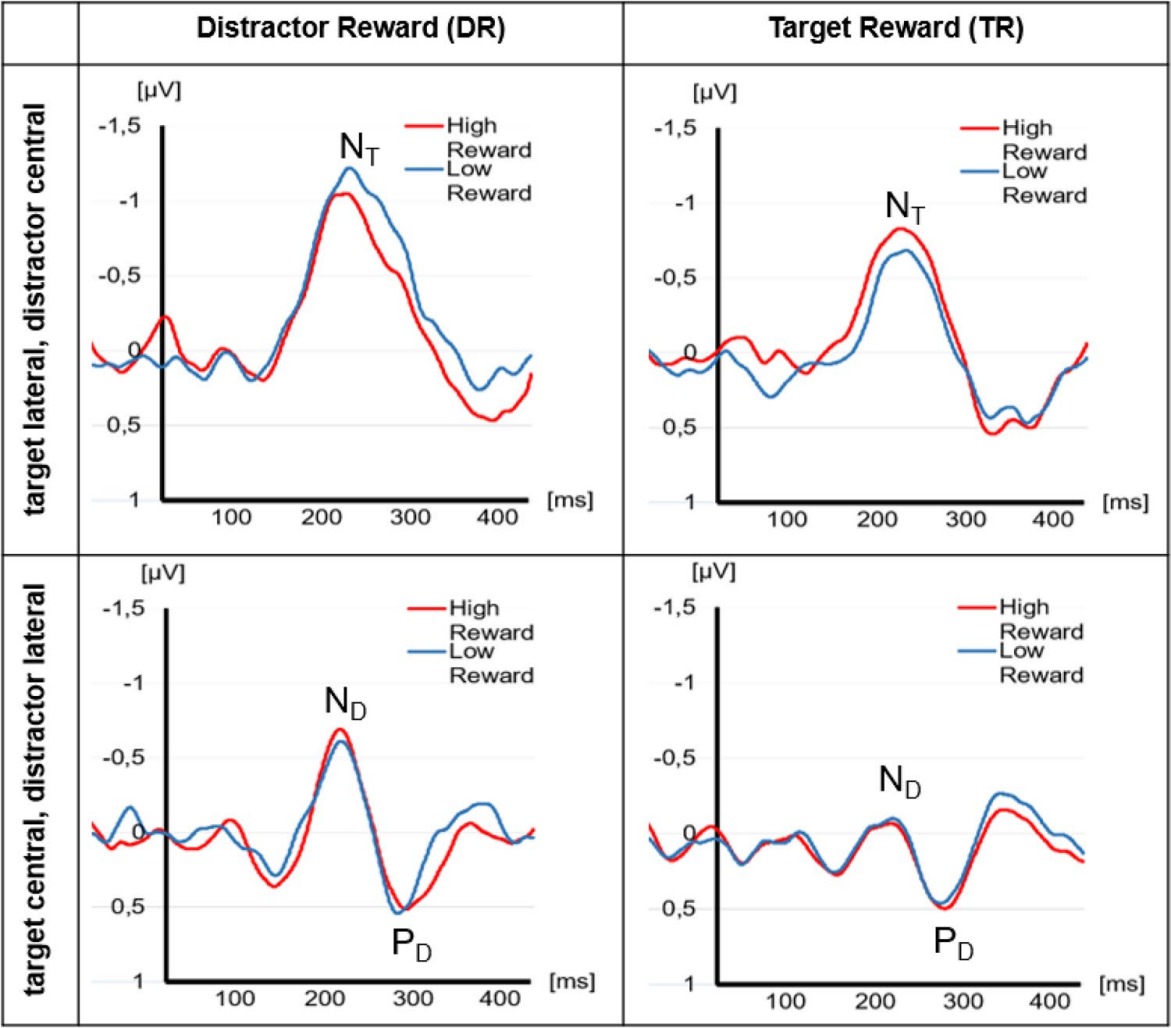
Grand average ERPs recorded in the distractor reward group (left) and in the target reward group (right). ERPs were pooled over the electrode pairs PO3/P7 and PO4/P8. The upper row shows the lateralized ERPs evoked by the search displays, when targets were presented at a lateral position and distractors were presented at a central midline position. For this, we calculated the difference waves contralateral–ipsilateral to target position. The lower row shows the lateralized ERPs evoked by the search displays, when targets were presented at a central midline position and distractors were presented at a lateral position. In this case, we calculated the difference waves contralateral–ipsilateral to distractor. The waves elicited by high-reward distractors/targets are presented in red, the waves elicited by low-reward distractors/targets in blue. The peaks of the N2pc subcomponents, target negativity (N_T_), distractor negativity (N_D_) and distractor positivity (P_D_), are marked

**Fig. 5:**
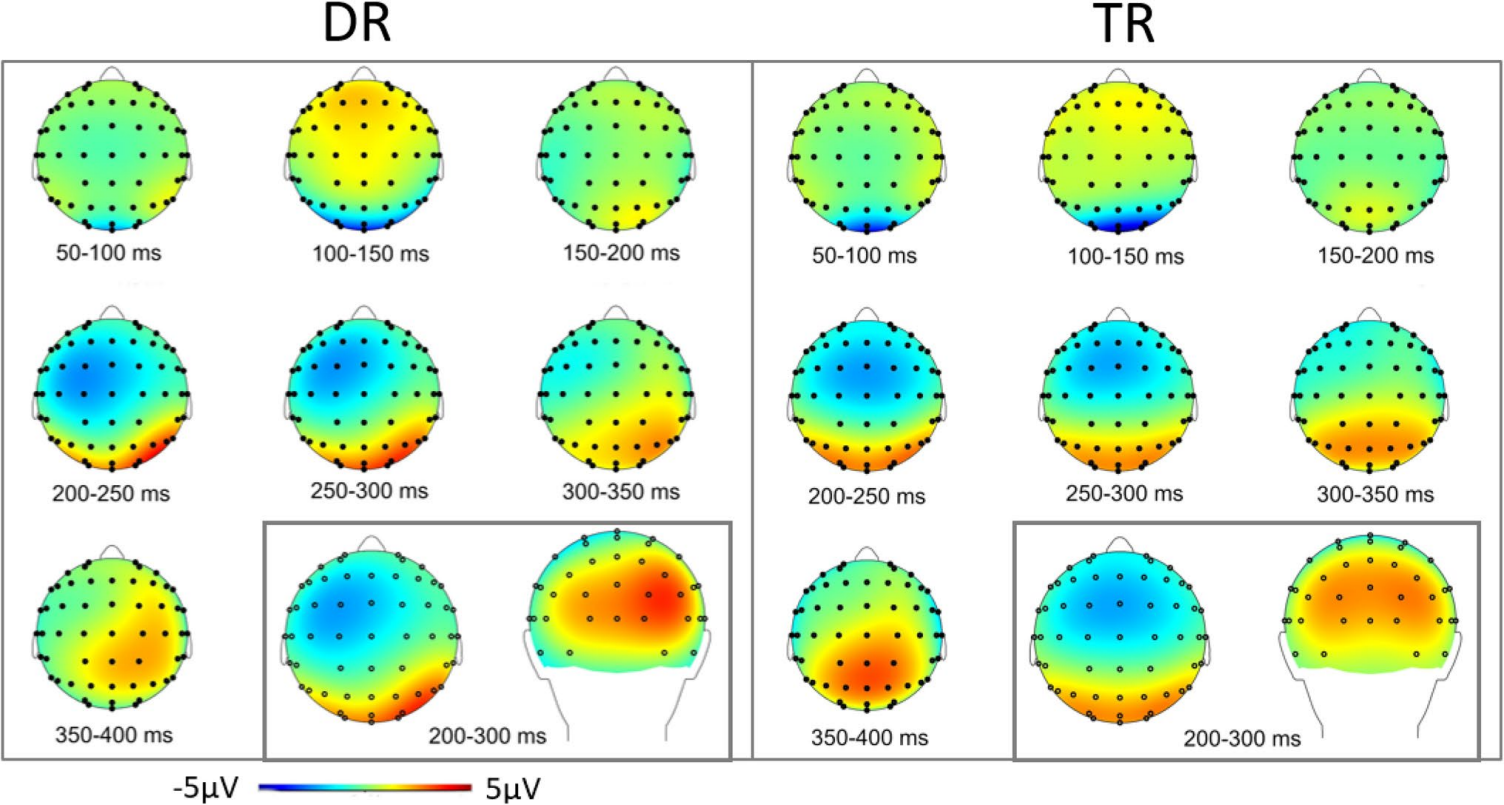
Scalp topographies obtained from the EEG data in the DR (left) and TR (right) group averaged within 50-ms—epochs between 50 and 400 ms after search display onset. The window in the bottom right corner shows the average signal within the time interval corresponding to the N2pc (200–300 ms after search display onset)

Tables 2 and 3 present the descriptive statistics for our ERP data in the DR and the TR group, respectively. Figure 3 shows the grand average ERP wavelines and Fig. 4 the lateralized ERPs recorded in the DR group and in the TR group for high and low reward trials. Additionally, Fig. 5 provides a topographic representation of the EEG signal. Below, we present the results for the N2pc subcomponents .

**Table 2:** Descriptive statistics for ERP data in the DR group

DR	N_T_: HR	N_T_: LR	N_D_: HR	N_D_: LR	P_D_: HR	P_D_: LR
N	20	20	20	20	20	20
Amplitude in µV	− 1.511	− 1.809	− 0.964	− 0.894	0.990	1.039
Std. Dev	0.842	0.756	0.853	0.677	0.970	0.963
Max	− 3.482	− 3.159	− 2.540	− 2.366	− 0.797	− 0.659
Min	0.124	− 0.680	0.642	0.258	4.000	3.849

*HR* high reward, *LR* low reward, *DR* distractor reward, *N*_T_ target negativity, *N*_D_  distractor negativity, *P*_D_ distractor positivity, *Std.Dev. * standard deviation

**Table 3:** Descriptive statistics for ERP data in the TR group

TR	N_T_: HR	N_T_: LR	N_D_: HR	N_D_: LR	P_D_: HR	P_D_:LR
N	20	20	20	20	20	20
Amplitude in µV	− 1.210	− 1.042	− 0.462	− 0.441	0.823	0.886
Std. Dev	0.636	0.655	0.540	0.453	0.367	0.457
Maximum	− 2.511	− 2.295	− 1.728	− 1.157	0.117	0.118
Minimum	− 0.119	− 0.039	0.854	0.339	1.521	1.667

*HR* high reward, *LR* low reward, *DR* distractor reward, *N*_T_ target negativity, *N*_D_  distractor negativity, *P*_D_ distractor positivity, *Std.Dev. * standard deviation

#### N_T_ Component

We found a significant group × reward condition interaction, *F*(1,38) = 6.736, *p* = 0.013, $\eta_{{\text{p}}}^{2}$ = 0.151. Thus, reward condition had a differential effect on N_T_ amplitude in DR and TR groups. Contrasts revealed that this interaction was driven by a significant difference between high and low reward trials in the DR group, *t*(38) = 2.219, *p* = 0.033 and no significant difference in the TR group. In addition, there was also a significant main effect of group, *F*(1,38) = 6.134, *p* = 0.014, $\eta_{{\text{p}}}^{2}$ = 0.149.

#### N_D_ Component

We found a significant main effect of group, *F*(1,38) = 6.093, *p* = 0.018, $\eta_{{\text{p}}}^{2}$ = 0.138. Thus, participants had significantly larger mean N_D_ amplitude in the DR than in the TR group.

#### P_D_ Component

No effect reached significance (*p* > 0.05).

### Source Space Analyses Results

Tables 4 and 5 (see supplementary material) list the current source density values gained from the averaged signal during a 50–400 ms time window after stimulus-onset, separately for the DR and the TR group. For each 50 ms epoch, the three sources with the highest current source density values were selected. Additionally, Table 6 (see supplementary material) lists the three sources with the highest current source density values obtained from the peaks in global field power.

On this descriptive level, it can be observed that for the DR group mainly occipital and temporal regions were involved in creating the EEG signal. The first peak was observed at 100 ms after search display onset and was located in occipital brain regions (cuneus, precuneus). The second peak was observed at 200 ms after search display onset and was also located in occipital regions (cuneus, middle occipital gyrus).

**Fig. 6:**
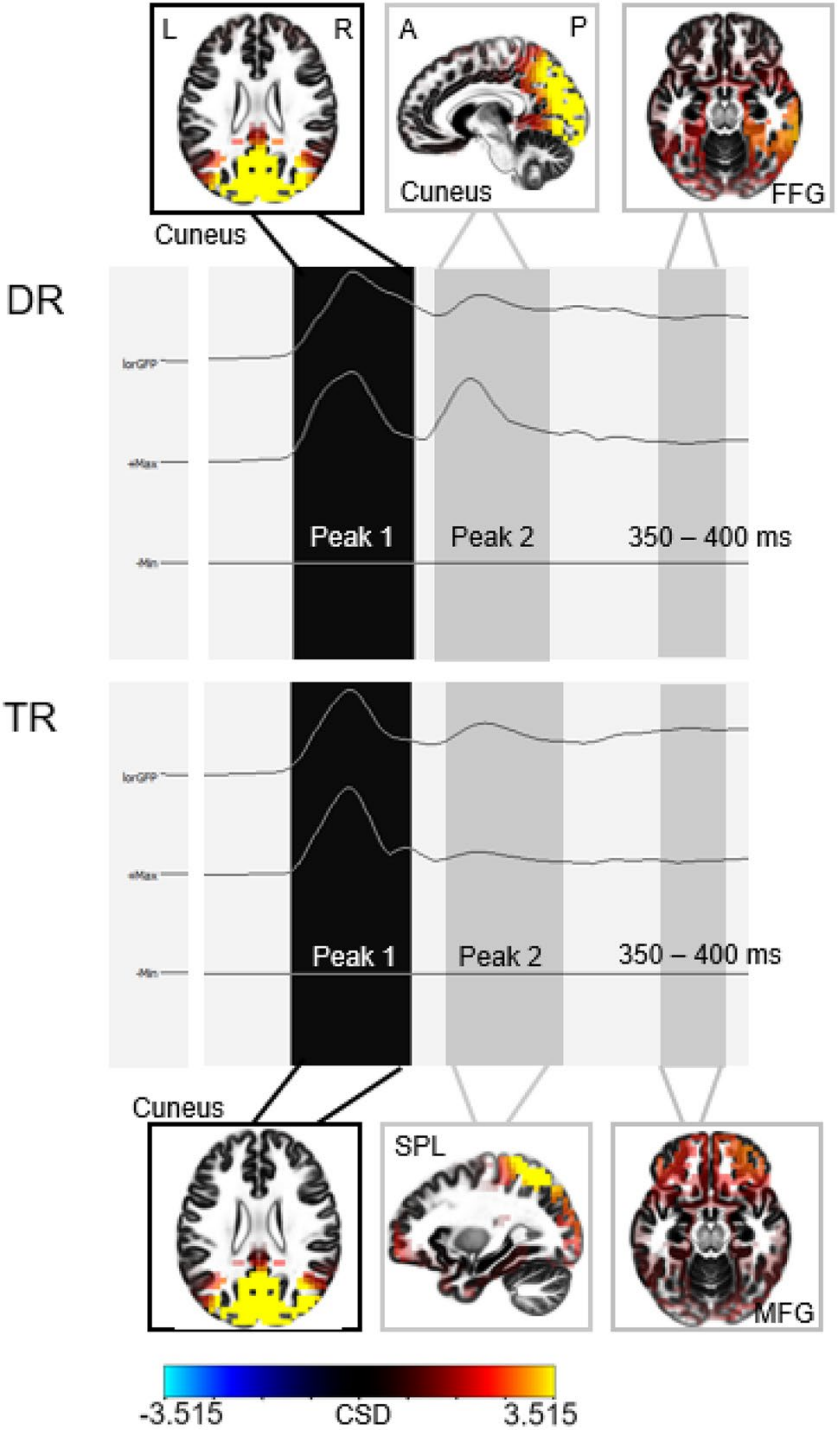
Visualization of the current source density values obtained from the EEG data in the DR (upper) and TR (lower) group over the course of 50–400 ms after search display onset. The highlighted time windows illustrate the regions with the highest current source density values at peak 1, peak 2 and from 350 to 400 ms after search display onset (*FFG* fusiform gyrus, *SPL* superior parietal lobule, *MFG* middle frontal gyrus)

For the TR group, early processing (50–150 ms after search display onset) comprised primarily occipital regions. As time progressed, inferior and superior parietal (150–300 ms) and frontal regions (350–400 ms) became involved. The first peak was observed at 100 ms after search display onset and was located in occipital brain regions (cuneus, precuneus). The second peak was observed at 210 ms after search display onset and was located in parietal regions (superior parietal lobule, postcentral gyrus, inferior parietal lobule). An illustration of the time course of current source density values can be seen in Fig. 6.

**Fig. 7:**
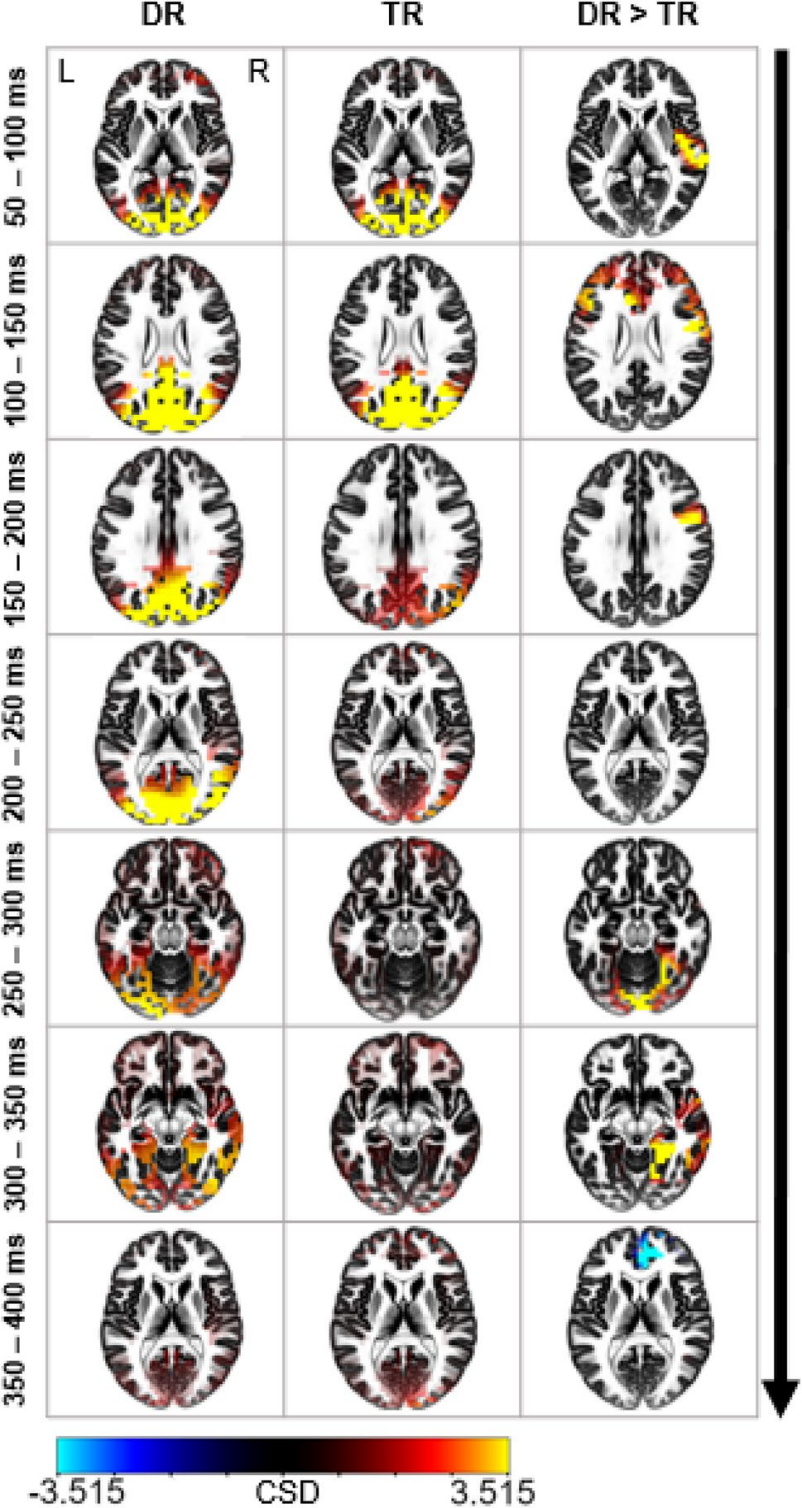
Illustration of the current source density values obtained from the EEG data within 50-ms-epochs after search display onset. The column on the left and the column in the middle show current source density values. The column on the right shows a visualization of the significant t-values in the contrast DR > TR (*p* < 0.05)

**Fig. 8:**
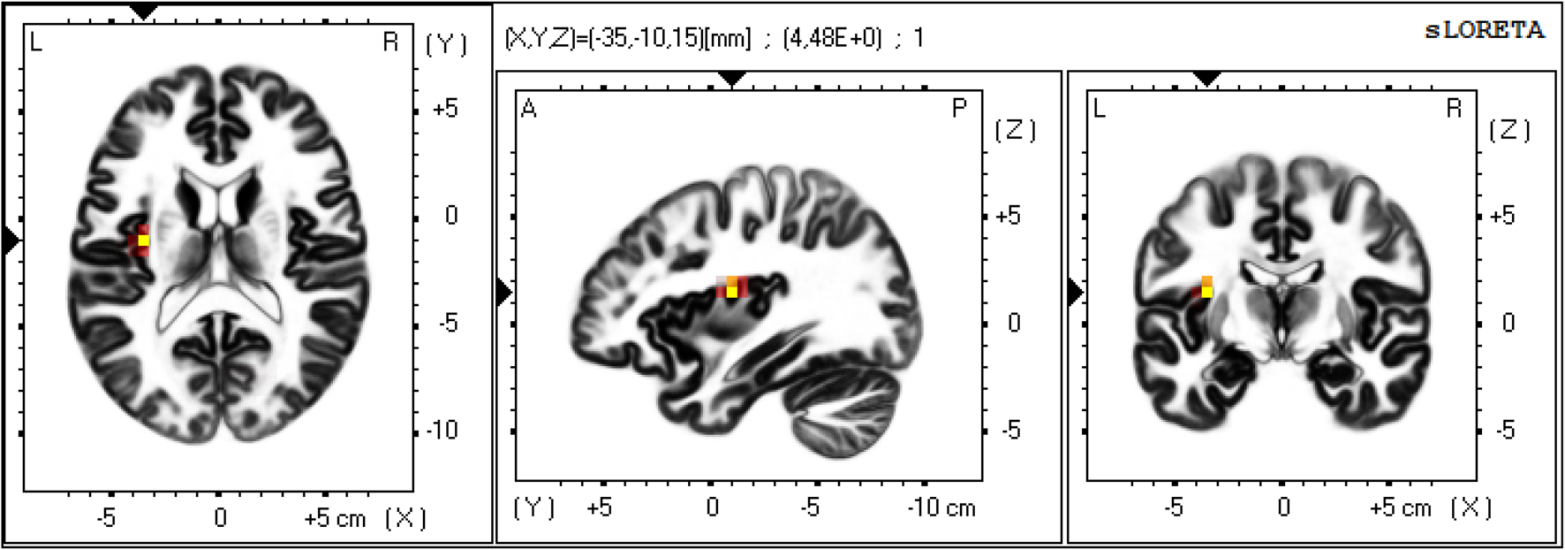
T-values of voxels in the contrast distractor reward, high reward > low reward for the time epoch corresponding to the P_D_ component. Significant voxels (*p* < 0.05) were located in the left insula (MNI-coordinates: − 35, − 10, 15, t-value = 4.483)

Furthermore, the results from a direct comparison of DR and TR groups (DR > TR) can be found in Table 7 (see supplementary material). Several parietal, temporal, limbic and frontal regions showed significantly (*p* < 0.05) more activation in the DR group, especially at early (50–200 ms after search display onset) and late (250–300 ms after search display onset) time intervals. At 350 ms after search display onset, the anterior cingulate, medial and superior frontal regions were significantly more activated in the TR than in the DR group. A visualization of this time course and the contrast between groups can be found in Fig. 7. Additionally, we discovered a significant difference between high and low rewards in the DR group (high reward > low reward) in the time interval corresponding to the P_D_ component of the ERP. The insula was significantly more involved for high rewards than for low rewards (*p* > 0.05, Fig. 8). No other comparisons of high and low-reward trials reached significance.

## Discussion

This study showed for the first time the differing timelines of attentional selection processes in the presence of reward-related targets and distractors in an Additional Singleton Task. When task-irrelevant distractors were associated with reward (DR group), the main neural sources were located in bilateral occipital cortex. At later time intervals (300–400 ms after search display onset), we also observed activation in temporal regions. On the other hand, when targets were associated with reward (TR group), occipital regions were involved at early time intervals (50–150 ms after search display onset), followed by inferior and superior parietal regions (150–300 ms after search display onset), occipital regions (300–350 ms after search display onset) and the frontal cortex (> 350 ms after search display onset). By directly contrasting distractor and target reward, we observed that associating rewards with task-irrelevant distractors increased activation in frontal, temporal, insular and cingulate regions early (100–200 ms) after search display onset. At later time intervals (250–400 ms), we found an increase in temporal and occipital activation for the DR group and enhanced frontal and anterior cingulate activation in the TR group.

Generally, it is believed that visual attention is controlled by two partially separated systems: the top-down system centered around dorsal posterior parietal and frontal cortex, dealing with cognitive selection of sensory information, and the bottom-up system centered around temporoparietal and ventral frontal cortex, dealing with the detection of salient and behaviorally-relevant events (Corbetta and Shulman 2002). It is assumed that bottom-up visual processing starts along the major visual pathways. The ventral pathway comprises V1, V2, V3, V4 and the inferior temporal cortex, and is mainly concerned with object- and feature-based processing. The dorsal pathway, on the other hand, is assumed to be concerned with spatial- and movement-related processing and comprises areas V1, V2, V3, middle temporal, superior temporal and parts of the posterior parietal cortex. From there, information is transmitted to the prefrontal cortex (Katsuki et al. 2014; Ungerleider and Haxby 1994). Woldorff et al. (2002), for example, showed that visual attention results in an early modulation of visual areas, with several returns of attention-related activations to these regions, indicating a more specific processing and analysis. Thus, the early occipital activation found in both groups (DR and TR) might reflect bottom-up attentional processing, extending into temporal and parietal regions as it moved along the visual pathways. However, recent research has demonstrated that bottom-up and value-based attention show substantial overlap in their underlying mechanisms (Anderson and Kim 2019). Both types of biases were associated with increased activation of the early visual cortex (Anderson et al. 2017). In the current study, corresponding occipital activation was found shortly (50 ms) after stimulus onset, but also at later time intervals (around 300 ms after search display onset in both groups). The direct contrast revealed that this later activation was stronger in the DR group, where the effect of reward was tied to the salient, but task-irrelevant distractor.

In the past, attentional capture by task-irrelevant distractors was associated with increased activation in parietal and frontal regions (de Fockert et al. 2004; Lavie et al. 2006). It was assumed that the frontal cortex and its top-down control functions would play an important role in resolving the competition between targets and attention-capturing distractors (Lavie et al. 2006). In our study, we found increased frontal activation for the DR group compared with the TR group. At early time intervals (100–200 ms after search display onset), several frontal areas showed enhanced activation when a reward-related distractor was present in contrast to when a reward-related target was present. This difference might be due to the stronger attentional capture effect induced by the rewarded distractor, which could also be observed in the EEG, where the N_D_ component (reflecting attentional capture) was significantly stronger in the DR than in the TR group. The stronger frontal activation in the DR group might also reflect a general increase in the need for top-down guidance, since in this group, the effect of reward was contrary to the task of the participant. However, at later time intervals (> 350 ms after search display onset), we found an increase in frontal activation for the TR group compared with the DR group. This increase was accompanied by a general increase in frontoparietal source activity in the TR group over the time course of the experiment. Since it was found that attentional mechanisms seem to be similar for different stimulus features (e.g. color or orientation; Girelli and Luck 1997), this effect should not be due to the fact that in the DR group reward was associated with the color of the distractor and in the TR group with the orientation of the target.

As already mentioned, reward-related attentional processing has been associated with activation in visual areas (Anderson et al. 2014, 2017; Garcia et al. 2018). Additionally, the caudate tail, the intraparietal sulcus (Anderson et al. 2014, 2017), as well as the anterior cingulate cortex (Hickey et al. 2010) were found to exhibit reward-driven activity. The anterior cingulate cortex is assumed to be deeply linked to the mesolimbic dopamine system. For example, it was proposed that dopaminergic influences can change perceptual representations of reward-related visual stimuli to make them more salient (Berridge and Robinson 1998). In the present study, we found anterior cingulate activation for both reward groups. However, for the DR group (compared to the TR group), this activation was stronger shortly after stimulus onset (100–150 ms), whereas in the TR group (compared to the DR group) the activation was stronger at the latest time interval (> 350 ms).

In addition to these results, we also found a significant difference between high and low-reward trials in the DR group during the time interval corresponding to the P_D_ EEG component, indicating distractor suppression (238–338 ms after search display onset). During this time interval, the left-hemispheric insula showed increased activation for high compared to low-reward trials. Insular activation has been associated with behavioral suppression (Haaranen et al. 2020; Lerner et al. 2009; Muhtadie et al. 2019), as well as with reward-based salience (Wang et al. 2019). The insula is assumed to project to the bottom-up salience network, enabling reward-related distractors to overcome inhibition in the visual cortex (Wang et al. 2019). Thus, the stronger activation of the left insula seems to reflect an increase in reward-related salience associated with high-reward distractors, which might induce a greater need for suppression compared with low-reward trials.

Studies investigating the time-courses of bottom-up and top-down reward, have shown that bottom-up effects generally occur earlier than top-down effects (Godijn and Theeuwes 2002; Meeter et al. 2010; Trappenberg et al. 2001). A similar difference was found with reward-associated stimuli. Preciado et al. (2017), for example, found that reward-related biases influenced attention early (see also MacLean and Giesbrecht 2015; Theeuwes and Belopolsky 2012; Failing et al. 2015; Nissens et al. 2020), but could be overcome by top-down control at later time points. In the current study, we found activation of visual regions, which have been associated with value-based attentional processes (Anderson et al. 2017), shortly after stimulus onset. In addition, reward-related distractors in comparison to reward-related targets increased activation in the anterior cingulate at 100 ms after search display onset. The insula, another brain region involved in reward-related processing, showed increased activation for high compared with low rewards at around 250 ms after stimulus presentation. Therefore, it can be concluded that reward modulations were already taking effect shortly after stimulus onset.

On the behavioral level, we observed a significant response time difference between high and low-reward trials in the TR group. This is in line with previous research (Kiss et al. 2009) and demonstrates that reward magnitude has had an influence on target processing. We did not observe a differential effect of high and low rewards on response times in the DR group. However, our ERP results showed that reward magnitude did reduce target prioritization in the DR group. This reduction of the N_T_ component in trials with high-reward distractors is in line with our expectations and with previous research (Feldmann-Wüstefeld et al. 2016). It implicates that target processing was impaired by concurrently presented high reward distractors. For the TR group, we expected the opposite effect, namely an increase in target prioritization for high-reward trials. Even though response time results and visual inspection of the ERP curve suggest that the effect might head in this direction, there was no significant difference between high and low-reward targets. Additionally, reward magnitude did not affect distractor processing, as reflected by N_D_ and P_D_ components.

The current study extends a growing literature dealing with the investigation of reward effects on attentional selection by presenting a first glance at the time courses of the underlying neural processes. Previous work has shown that reward can increase the efficiency of target selection, when targets were associated with reward (Engelmann et al. 2009; Della Libera and Chelazzi 2006), while at the same time decreasing the efficiency of target selection, when distractors were associated with reward (Anderson and Yantis 2013; Hickey et al. 2010; Chelazzi et al. 2013; Le Pelley et al. 2015; Bourgeois et al. 2017; Watson et al. 2019). In the present study, we showed that these two reward-dependent processes were associated with differences in the time course of neural activation. Rewarding distractors compared to rewarding targets led to an increase in frontal activation at early time intervals, indicating stronger top-down control due to an enhanced attentional capture effect by the rewarded distractor. Both groups increased activation in reward-related regions, such as the visual cortex and the anterior cingulate. However, the activation in the anterior cingulate was stronger and occurred earlier in the distractor-reward group compared to the target-reward group. These time-dependent differences might indicate that the neural mechanisms underlying reward biasing could be different for task-relevant and task-irrelevant stimuli. Additionally, the insula was found to show activation during suppression of reward-related distractors, reinforcing its important role in value-driven attention. Further studies may address the validation of the current localization results using simultaneous EEG-fMRI (Esposito et al. 2009) and the assessment of the causality of the involved brain regions for behavior using non-invasive brain stimulation (Elyamany et al. 2020).

## Supplementary Information

Below is the link to the electronic supplementary material.

Supplementary file1 (DOCX 45 KB)
