# Supplementary material for: Differing Time Courses of Reward-Related Attentional Processing: An EEG Source-Space Analysis
Source: Brain Topogr. 2021 Mar 18;34(3):283–96. doi: 10.1007/s10548-021-00827-3 (PMC8099853; doi:10.1007/s10548-021-00827-3)
Supplement: Supplementary file 1 — Supplementary file1 (DOCX 45 KB) [file 10548_2021_827_MOESM1_ESM.docx]

**Appendix**

**Supplementary Material**

**Table 4.** The three regions with the highest current source density values obtained from the averaged signal in the DR group within 50 ms epochs after stimulus onset (averaged across

participants and reward conditions).

| DR | 50-100 ms | 100-150 ms | 150-200 ms | 200-250 ms | 250-300 ms | 300-350 ms | 350-400 ms |
| --- | --- | --- | --- | --- | --- | --- | --- |
| Region 1 | Cuneus (R) | Cuneus (L) | Cuneus (L) | Cuneus (L) | Cuneus (L) | FFG (R) | FFG (R) |
| MNI coord. | 5, -95, 25 | -5, -95, 25 | -10,  -100, 20 | -10,  -100, 20 | -10, -100, 20 | 45, -60, -25 | 55, -40, -30 |
| CSD in μAmm/mm^3^ | 10.497 | 8.567 | 8.820 | 9.002 | 4.890 | 4.126 | 3.515 |
| Region 2 | Cuneus (L) | Cuneus (R) | MOG (L) | MOG (L) | Cuneus (R) | ITG (R) | MTG (R) |
| MNI coord. | -5, -95, 25 | 5, -95, 25 | -10,  -100, 10 | -15,  -100, 10 | 5, -90, 35 | 50, -55, -20 | 65, -35, -20 |
| CSD in μAmm/mm^3^ | 10.451 | 7.924 | 8.190 | 7.936 | 4.634 | 3.745 | 3.428 |
| Region 3 | MOG (R) | Precuneus (L) | Cuneus (R) | Cuneus (R) | Precuneus (R) | IOG (R) | ITG (R) |
| MNI coord. | 10, -100, 10 | -10, -85, 40 | 5, -100, 15 | 5, -95, 25 | 15, -85, 40 | 40, -85, -20 | 60, -35, -25 |
| CSD in μAmm/mm^3^ | 8.616 | 7.512 | 7.821 | 7.218 | 4.585 | 3.685 | 3.421 |

*MNI coord. = MNI coordinates (x,y,z), ITG = inferior temporal gyrus, MTG = middle temporal gyrus, MOG = middle occipital gyrus, FFG = fusiform gyrus, CSD = current source density, L= left, R = right.*

**Table 5.** The three regions with the highest current source density values obtained from the averaged signal in the TR group within 50 ms epochs after stimulus onset (averaged across participants and reward conditions).

| TR | 50-100 ms | 100-150 ms | 150-200 ms | 200-250 ms | 250-300 ms | 300-350 ms | 350-400 ms |
| --- | --- | --- | --- | --- | --- | --- | --- |
| Region 1 | Cuneus (L) | Cuneus (R) | IPL (R) | SPL (R) | SPL (R) | Cuneus (L) | MFG (R) |
| MNI coord. | -15, -95, 30 | 15, -95, 25 | 45, -70, 45 | 30, -65, 60 | 35, -60, 60 | -15, -95, 30 | 35, 55,  -15 |
| CSD in μAmm/mm^3^ | 1.068 | 9.127 | 3.912 | 4.644 | 3.117 | 3.175 | 2.929 |
| Region 2 | Cuneus (R) | Cuneus (L) | SPL (R) | Postcen. (R) | Postcen. (R) | Precuneus (L) | SFG (R) |
| MNI coord. | 5, -95, 25 | -5, -90, 30 | 45, -65, 50 | 25, -55, 70 | 25, -55, 70 | -15, -85, 40 | 30, 55,  -15 |
| CSD in μAmm/mm^3^ | 1.034 | 8.048 | 3.819 | 4.396 | 3.114 | 2.999 | 2.890 |
| Region 3 | Precuneus (L) | Precuneus (R) | Precuneus (R) | IPL (R) | IPL (R) | Cuneus (R) | IFG (R) |
| MNI coord. | -15, -85, 40 | 10, -85, 40 | 40, -75, 40 | 40, -55, 60 | 40, -55, 60 | 10, 100, 15 | 25, 35,  -25 |
| CSD in μAmm/mm^3^ | 0.9663 | 7.952 | 3.645 | 4.291 | 3.007 | 2.986 | 2.8779 |

*MNI coord. = MNI coordinates (x,y,z), MFG = middle frontal gyrus, IFG = inferior frontal gyrus, SFG = superior frontal gyrus, IPL = inferior parietal lobule, Postcen. = postcentral gyrus, SPL = superior parietal lobule, CSD = current source density, L= left, R = right.*

**Table 6.** The three source regions with the highest current source density values obtained from the averaged signal in the DR and the TR group within ±50 ms epochs around the peaks in global field power (averaged across participants and reward conditions).

|  | DR | | TR |  |
| --- | --- | --- | --- | --- |
| Peak | **Peak 1** | **Peak 2** | **Peak 1** | **Peak 2** |
| TF | 100 (50 - 150) ms | 200 (150 - 250) ms | 100 (50 - 150) ms | 210 (160 - 260) ms |
| Region 1 | Cuneus (L) | Cuneus (L) | Cuneus (R) | SPL (R) |
| MNI coordinates | -5, -95, 25 | -10, -100, 20 | 15, -95, 25 | 30, -65, 60 |
| CSD in μAmm/mm^3^ | 9.450 | 8.855 | 9.905 | 4.212 |
| Region 2 | Cuneus (R) | MOG (L) | Cuneus (L) | Postcentral Gyrus (R) |
| MNI coordinates | 5, -90, 30 | -15, -100, 10 | -5, -90, 30 | 25, -55, 70 |
| CSD in μAmm/mm^3^ | 8.828 | 8.004 | 9.138 | 3.993 |
| Region 3 | Precuneus (R) | Cuneus (R) | Precuneus (R) | IPL (R) |
| MNI coordinates | 5, -85, 40 | 5, -100, 10 | 10, -85, 40 | 40, -55, 60 |
| CSD in μAmm/mm^3^ | 7.722 | 7.270 | 8.613 | 3.889 |

*MNI coordinates (x,y,z), SPL = superior parietal lobule, MOG = middle occipital gyrus, IPL = inferior parietal lobule, CSD = current source density, L = left, R = right, TF = time frames.*

**Table 7.** T-values and MNI-coordinates of significant voxels in the contrast DR > TR for 50-ms-epochs after search display onset (*p* < 0.05).

| DR vs TR | 50-100 ms | 100-150 ms | 150-200 ms | 200-250 ms | 250-300 ms | 300-350 ms | 350-400 ms |
| --- | --- | --- | --- | --- | --- | --- | --- |
| SFG (L) |  | -35, 50, 25 |  |  |  |  |  |
| t-value |  | 4.109 |  |  |  |  |  |
| SFG (R) |  | 25, 30, 50 |  |  |  |  | 20, 65,  -15 |
| t-value |  | 3.990 |  |  |  |  | -3.799 |
| MFG  (L) |  | -45, 20, 25 |  |  |  |  |  |
| t-value |  | 4.858 |  |  |  |  |  |
| MFG (R) |  | 45, 0, 40 | 45, 0, 40 |  | 30, 25,  -50 |  |  |
| t-value |  | 6.132 | 4.596 |  | 3.833 |  |  |
| IFG (L) |  | -50, 25, 25 |  |  |  |  |  |
| t-value |  | 4.706 |  |  |  |  |  |
| IFG (R) |  | 45, 0, 35 | 55, 0, 25 |  |  |  |  |
| t-value |  | 6.338 | 5.247 |  |  |  |  |
| MedFG (L) |  | -15, 25, 35 |  |  |  |  |  |
| t-value |  | 4.524 |  |  |  |  |  |
| MedFG (R) |  | 5, 30, 35 |  |  |  |  | 10, 50, 10 |
| t-value |  | 4.249 |  |  |  |  | -3.917 |
| ACin. (L) |  | -10, 20, 25 |  |  |  |  |  |
| t-value |  | 5.760 |  |  |  |  |  |
| ACin. (R) |  | 5, 20, 25 |  |  |  |  | 10, 45, 10 |
| t-value |  | 3.863 |  |  |  |  | -4.006 |
| PCin. (L) |  |  |  |  | -10, 20, 25 |  |  |
| t-value |  |  |  |  | 5.760 |  |  |
| PCin. (R) |  |  |  |  |  | 20, -55, 5 |  |
| t-value |  |  |  |  |  | 3.778 |  |
| Cingulate (L) |  | -10, 15, 30 |  |  |  |  |  |
| t-value |  | 5.354 |  |  |  |  |  |
| Cingulate (R) |  | -10, 15, 30 |  |  |  |  |  |
| t-value |  | 5.354 |  |  |  |  |  |
| Parahipp. (R) |  |  |  |  | 25, -50,  -15 | 25, -55,  -10 |  |
| t-value |  |  |  |  | 4.569 | 4.502 |  |
| Insula (L) |  | -30, 20, 15 |  |  |  |  |  |
| t-value |  | 5.668 |  |  |  |  |  |
| Insula (R) | 45, -10, 10 | 45, -10, 15 | 40, 0, 20 |  |  |  |  |
| t-value | 3.600 | 3.802 | 4.452 |  |  |  |  |
| Precen. (L) |  | -40, 25, 40 |  |  |  |  |  |
| t-value |  | 3.793 |  |  |  |  |  |
| Precen. (R) |  | 50, 0, 35 | 50, -5, 35 |  |  |  |  |
| t-value |  | 6.465 | 5.366 |  |  |  |  |
| Postcen. (R) |  | 65, -10, 25 | 60, -10, 20 |  |  |  |  |
| t-value |  | 4.345 | 3.615 |  |  |  |  |
| SPL (L) |  |  |  |  | -80, 45,  -25 |  |  |
| t-value |  |  |  |  | 3.768 |  |  |
| STG (R) | 65, -25, 0 | 65, -20, 0 | 65, -5, 10 |  | 5, -5, 59 |  |  |
| t-value | 3.704 | 4.216 | 4.030 |  | 3.732 |  |  |
| MTG (R) | 65, -30, 0 | 65, -20,  -5 |  |  | 45, -65,  -5 | 45, -60,  -5 |  |
| t-value | 3.753 | 4.210 |  |  | 4.02 | 3.853 |  |
| ITG (R) |  | 60, -55,  -15 |  |  | 50, -75,  -5 |  |  |
| t-value |  | 3.691 |  |  | 3.918 |  |  |
| FFG (L) |  |  |  |  | -20, -90, -25 |  |  |
| t-value |  |  |  |  | 4.361 |  |  |
| FFG (R) |  | 50, -45,  -15 |  |  | 25, -55,  -15 | 25, -55,  -15 |  |
| t-value |  | 3.603 |  |  | 4.490 | 4.490 |  |
| Trans. (R) |  | 60, -10, 15 |  |  |  |  |  |
| t-value |  | 3.904 |  |  |  |  |  |
| LG (L) |  |  |  |  | -5, -90,  -20 |  |  |
| t-value |  |  |  |  | 5.078 |  |  |
| LG (R) |  |  |  |  | 5, -90,  -20 | 20, -60,  -5 |  |
| t-value |  |  |  |  | 5.122 | 4.330 |  |
| Precuneus (L) |  |  |  |  | -75, 15,  -20 |  |  |
| t-value |  |  |  |  | 3.868 |  |  |
| Cuneus (L) |  |  |  |  | -25, -80, 15 |  |  |
| t-value |  |  |  |  | 4.012 |  |  |
| Cuneus (R) |  |  |  |  | 10, -85, 15 |  |  |
| t-value |  |  |  |  | 4.007 |  |  |
| MOG (L) |  |  |  |  | -30, -80, 15 |  |  |
| t-value |  |  |  |  | 4.021 |  |  |
| MOG (R) |  |  |  |  | 25, -85,  -15 |  |  |
| t-value |  |  |  |  | 3.965 |  |  |
| IOG (L) |  |  |  |  | -90, -20, -25 |  |  |
| t-value |  |  |  |  | 3.899 |  |  |
| IOG (R) |  |  |  |  | 40, -75,  -10 |  |  |
| t-value |  |  |  |  | 4.101 |  |  |

*MNI coordinates (x,y,z), IOG = inferior occipital gyrus, MOG = middle occipital gyrus, FFG = fusiform gyrus, Precen. = precentral gyrus, Postcen. = postcentral gyrus, ACin. = anterior cingulate gyrus, PCin. = posterior cingulate gyrus, SFG = superior frontal gyrus, MFG = middle frontal gyrus, MedFG = medial frontal gyrus, IFG = inferior frontal gyrus, MTG = middle temporal gyrus, ITG = inferior temporal gyrus, STG = superior temporal gyrus, SPL = superior parietal lobule, Trans. = transverse temporal gyrus, Parahipp. = parahippocampal gyrus, LG = lingual gyrus, L = left, R = right.*
